# Relationships between health outcomes in older populations and urban green infrastructure size, quality and proximity

**DOI:** 10.1186/s12889-020-08762-x

**Published:** 2020-05-06

**Authors:** Matthew Dennis, Penny A. Cook, Philip James, C. Philip Wheater, Sarah J. Lindley

**Affiliations:** 1 Department of Geography, School of Environment Education and Development, University of Manchester, Arthur Lewis Building, Manchester, M13 9PL UK; 2 School of Health and Society, University of Salford, The Crescent, Allerton Building, Salford, M5 4WT UK; 3 School of Science, Engineering and Environment, University of Salford, The Crescent, Peel Building, Salford, M5 4WT UK; 4 School of Science and the Environment, Manchester Metropolitan University, Chester Street, John Dalton Building, Manchester, M1 5GD UK

## Abstract

**Background:**

There is a growing body of literature supporting positive associations between natural environments and better health. The type, quality and quantity of green and blue space (‘green-space’) in proximity to the home might be particularly important for less mobile populations, such as for some older people. However, considerations of measurement and definition of green-space, beyond single aggregated metrics, are rare. This constitutes a major source of uncertainty in current understanding of public health benefits derived from natural environments. We aimed to improve our understanding of how such benefits are conferred to different demographic groups through a comprehensive evaluation of the physical and spatial characteristics of urban green infrastructure.

**Methods:**

We employed a green infrastructure (GI) approach combining a high-resolution spatial dataset of land-cover and function with area-level demographic and socio-economic data. This allowed for a comprehensive characterization of a densely populated, polycentric city-region. We produced multiple GI attributes including, for example, urban vegetation health. We used a series of step-wise multi-level regression analyses to test associations between population chronic morbidity and the functional, physical and spatial components of GI across an urban socio-demographic gradient.

**Results:**

GI attributes demonstrated associations with health in all socio-demographic contexts even where associations between health and overall green cover were non-significant. Associations varied by urban socio-demographic group. For areas characterised by having higher proportions of older people (‘older neighbourhoods’), associations with better health were exhibited by land-cover diversity, informal greenery and patch size in high income areas and by proximity to public parks and recreation land in low income areas. Quality of GI was a significant predictor of good health in areas of low income and low GI cover. Proximity of publicly accessible GI was also significant.

**Conclusions:**

The influence of urban GI on population health is mediated by green-space form, quantity, accessibility, and vegetation health. People in urban neighbourhoods that are characterised by lower income and older age populations are disproportionately healthy if their neighbourhoods contain accessible, good quality public green-space. This has implications for strategies to decrease health inequalities and inform international initiatives, such as the World Health Organisation’s Age-Friendly Cities programme.

## Background

Exposure to the natural environment has long been recognised as an important component of human well-being. The continued expansion of urban areas and sustained rural-to-urban migration have stimulated recent research on the role that green-space might play in urban planning and policy in a public health context [1]. In line with demographic projections and increased urbanization, the prevalence of chronic morbidity is expected to increase and green-space in cities presents one means to help reduce a range of chronic disorders related to modern urban lifestyles including obesity [2], depression [3], cardio-vascular illness and diabetes [4, 5]. However, despite the unique and complex environments in which urban residents live [6], the majority of epidemiological studies exploring the relationship between green-space and health have focused on socio-economic rather than physical environment characteristics in attempts to illuminate the inconsistent findings reported in the literature [4, 7–10]. Such work has neglected the complex character of the natural environment, its various physical characteristics and its spatial distribution in relation to socio-demographic gradients. For example, previous studies exploring associations between the natural environment and human health in urban areas have typically employed simplified metrics such as proportion of green-space cover or the Normalised Difference Vegetation Index (NDVI) [7–10]. In contrast, a ‘green infrastructure’ (GI) approach provides a sounder basis for understanding relationships between the natural environment and human health and wellbeing. This is because the concept of green infrastructure, in contrast to ‘green-space’ moves beyond simplistic catch-all metrics. A GI perspective considers how other quantity and quality-related considerations such as the form (land-cover e.g. grass, trees, water), function (e.g. parks, gardens, agriculture), spatial arrangement (e.g. connectivity and diversity) and socio-economic context of green-blue features in the urban landscape contribute to healthy, sustainable environments ([11, 12] ). Such characteristics are relevant to how people experience the natural environment and the term GI helps to recognise the multiple direct and indirect benefits derived from the functions provided by diverse but interconnected ‘green’ and ‘blue’ elements in cities, such as urban parks, waterways, gardens and tree canopy [13]. These ecosystem benefits (well-being benefits afforded to people through the presence of GI and GI-related processes) related to land-use and land-cover types may influence different socio-demographic groups as a function of their geographical distribution [14]. For example, socio-economic groups that spend more time closer to the home environment, such as stay-at-home parents and some older people, appear to benefit disproportionately from nearby nature [9]. In addition, recent research demonstrates that the ability of certain land-cover components to improve urban environmental conditions varies according to land-use. The alleviation of heat stress by canopy cover has been shown to be greater for trees in domestic gardens than those in parks as a function of their spatial distribution [15]. Similarly, informal spaces such as street greenery and roadside vegetation may reduce surface temperatures [ibid], to which older populations are more sensitive [16], highlighting their importance for neighbourhoods associated with particular demographic groups.

A recent review by Zhang et al. [17] suggests an emphasis on quantity rather than quality of green-space in epidemiological research to date with few studies attempting to incorporate measures of both. Despite the current availability of a wealth of open-source environmental data, the application of comprehensive, detailed characterizations of urban GI to the analysis of ill-health at an appropriate resolution is yet to be delivered. More recently, Dennis et al. [18] demonstrated the possibility of achieving fine-grain (10 m resolution), thematically detailed representations of urban GI through a comprehensive characterization of land-cover (the physical form that GI takes, e.g. grass, trees, water), land-use (the function associated with areas of GI, e.g. parks, gardens, institutional land) and landscape properties (the spatial characteristics of GI components e.g. the size and connectedness of patches of green space) using geospatial data and GIS and remote-sensing techniques. Such measures are a necessary step given the complex character of urban environments where exposure to the natural environment is moderated according to land-use (i.e. function), land-cover (i.e. form) and ownership [19]. Previous epidemiological studies have suffered from poor spatial and/or thematic precision in their measures of green space. Typically these have employed mid to low resolution data (i.e. >30m) for the calculation of vegetation indices [e.g. [20–23]. Similarly, coarse characterisations of urban land-use such as the European-level Corine or UK Land Cover Map data sets that group all urban areas into one or two broad categories are common (see [18] for a discussion of these and other available data).

We argue that operationalizing a GI approach through the use of spatially and thematically detailed data sets, encompassing the range of urban landscape characteristics pertinent to health and wellbeing, is needed to advance the field towards more robust implementations of nature-based public health interventions. To this end, our study employs a suite of social and physical environmental variables in order to build a comprehensive picture of links between characteristics of urban green infrastructure and local health status to fill an important science-policy gap. We predicted that GI should exhibit varying associations with health as a function of land-use and land-cover attributes, and socio-demographic context. For example, the presence of local accessible GI may provide particular benefit to the least physically mobile populations. Given that physical mobility can be constrained in both older and more economically deprived groups, members of the population falling into both of these categories should logically rely most strongly on nearby green infrastructure for nature experience. Moreover, such sectors of the population also tend to be the most vulnerable to climate-related hazards for which the presence of GI is a mitigating factor [22]. Related to the latter, plant vigour (a measure of healthy growing vegetation) underpins the performance of GI and its ability to deliver urban-relevant ecosystem benefits (such as heat stress mitigation [23] and filtering of air-borne particulates [24]). Given that economically deprived areas also often contain lowest overall GI cover [25] the quality (vigour) of existing vegetation may be disproportionately significant in these vulnerable areas from the perspective of GI-related health benefits. Based on these assumptions we developed three hypotheses. Firstly, we supposed that areas with a higher proportion of older people, especially those on low income, should benefit disproportionately from natural features in the vicinity of the home such as domestic gardens, nearby parks, informal neighbourhood greenery and the presence of urban trees. A second hypothesis was posited that the quality of GI (vegetation vigour as an indicator of heathy biological function) is a significant consideration in GI performance and its potential to confer health benefits to people, especially in areas of low overall GI-cover. Lastly, we explored the assumption that GI attributes related to landscape properties such as diversity, connectivity and patch size, as characteristics which promote landscape resilience, green-space access and the delivery of ecosystem benefits might exert a unique influence on associations between GI and health [26, 27].

## Methods

### Green infrastructure data

**Fig. 1:**
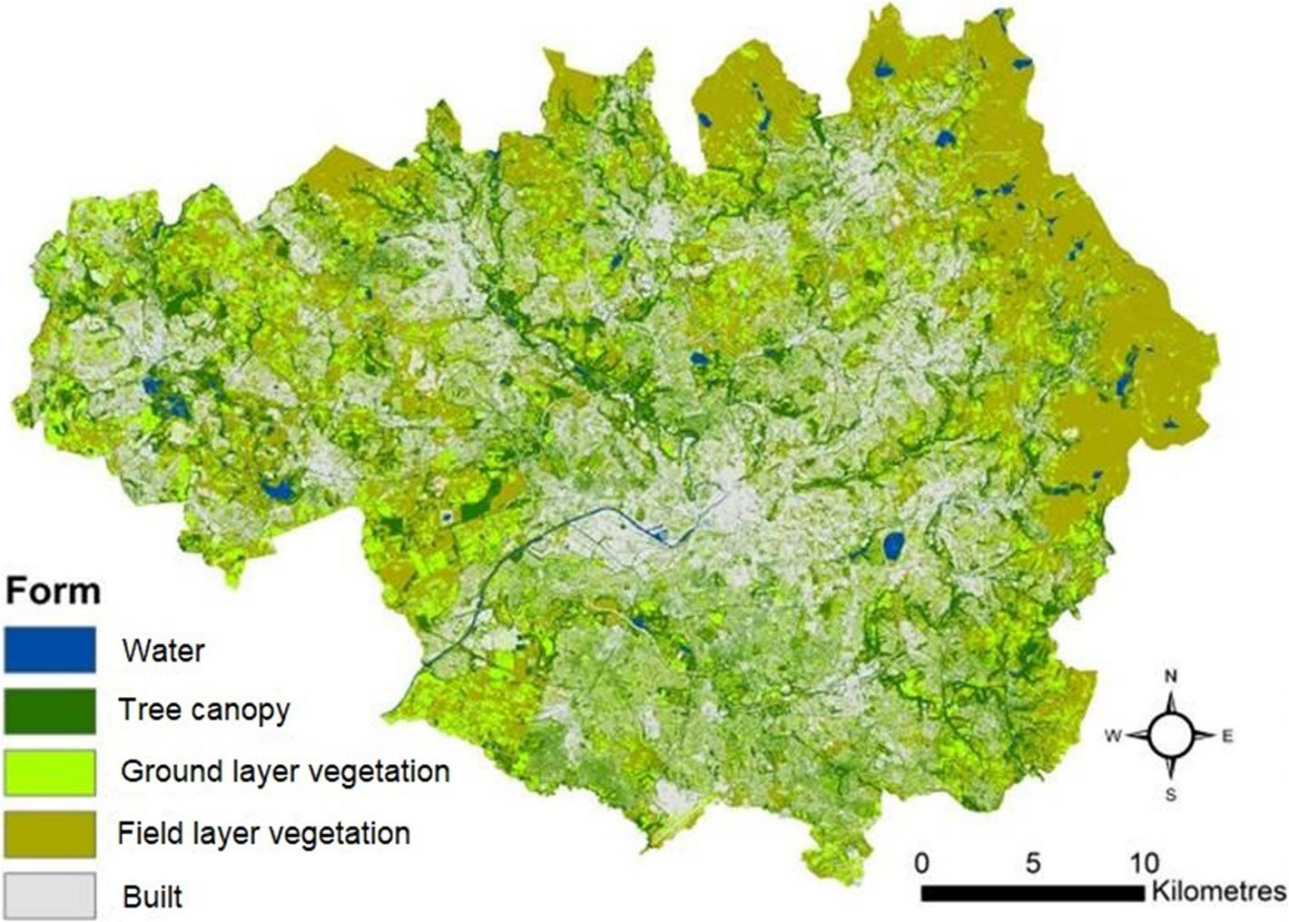
Land-cover representation of the Greater Manchester city region ([18]: contains City of Trees (2011) and European Space Agency (2016) data)

Spatial data were obtained from a novel landscape dataset representing urban green infrastructure (Integrated Landscape Map: ILM) [18]. These data cover the Greater Manchester city region, an urban area of 1,276 km^2^ with a population of 2.8 million as of 2016 [18]. The dataset provides high (10 m) resolution, spatially co-incident information on land-use and land-cover. The ILM combines remotely sensed data on vegetation and water cover (classified from Sentinel 2A, 2016 data) [28], digitized tree canopy data [29] and land-use data from Ordnance Survey [30–32]. Figure 1 shows the land-cover derived from this dataset.

### Assessment of GI quantity

Green land-use in the ILM dataset includes public parks and recreation (recreational spaces such as parks, playing fields, allotments and other sports facilities), amenity land (landscaped open spaces in residential or commercial areas with primarily aesthetic functions), private gardens, institutional land, previously-developed land, peri-urban and informal urban greenery (street trees, road verges and other green and blue land-cover not associated with formal green-space types). Land-cover is characterised as built, ground layer vegetation (grass lawns and other ground flora), field layer vegetation (forbs and shrubs), tree canopy, and water. Percentage cover by individual land-uses and land-covers were calculated for each of the 1673 Lower Super Output Areas (LSOAs) neighbourhoods within Greater Manchester in ArcGIS 10.4.1 [33]. Total green land-cover LSOAˉ^1^ ranged from 0.62 % to 98.81 % with a mean of 57.45% (SD 19.54). LSOAs are census reporting units for small area statistics in England and Wales with a mean population of 1666 persons for which socio-economic data were also obtained [34].

Built-cover within land-uses otherwise considered as green infrastructure has been highlighted as a considerable problem for the ability of GI to provide ecosystem benefits. Recently, work by Baker et al. exposed the significant degree of built cover found in UK domestic gardens (on average 50%) [35]. Elsewhere, studies on garden size and provision per capita suggest that domestic gardens have a significant positive influence on population health, greater than that implied by public green-spaces [36, 37]. It is unclear however to what extent non-built cover in gardens and other urban green land-use types may mediate these health outcomes. We were able to include such consideration in our analysis as the ILM dataset [18] permitted the calculation of cover by land-use as well as percentage non-built cover of gardens, institutional land, amenity and public parks and recreation classes (i.e. all major urban land-uses associated with GI) per LSOA.

### Assessment of GI quality

A range of metrics underpinning GI performance (connectivity, diversity and vegetation health, [38] were calculated to reflect GI quality in addition to the more standard percentage cover measures. Landscape indices related to land-cover diversity (Shannon’s Index: SHDI), mean patch size and connectivity (effective mesh size: Meff (see Jaeger [39]) were calculated, based on green land-cover, for each LSOA using the QGIS plug-in LecoS 2.0.7 [40]. Mean GI patch size is calculated as the mean area (in m^2^ of patches of non-built land-cover) and the metric effective mesh size (Meff) provides a measure of the total area of connected GI in a given area. In addition, patch size of individual land-uses was determined through OS MasterMap data [32]. Connectivity here is defined as the probability that two randomly selected points in the landscape (in our case in each LSOA) will be located within the same contiguous non-built patch (and therefore connected). This probability value (i.e. 0 to 1) is then multiplied by the total area of the landscape to give a final value for Meff (in m^2^).

A measure of vegetation health (or vigour), was also obtained by isolating pixels classed as green-cover in the ILM and calculating the mean Normalized Difference Vegetation Index LSOAˉ^1^ (NDVI: Summer, 2016) values. This vegetation-specific NDVI (vNDVI) was used based on the assumption that land-cover with a higher level of vigour indicates healthy and well maintained vegetation. Whereas mean NDVI values, calculated for example at the neighbourhood level, are commonly employed elsewhere in epidemiological studies as a measure of greenness, the metric, used in this way, is more closely associated with vegetation density and therefore a metric of relative abundance. However by isolating pixels classified as vegetation in our dataset, and using this layer as a mask in our NVDI calculation, our aim was to delineate the greenness of individual patches of vegetation as a measure of GI health. At the LSOA level, mean vNDVI was only weakly correlated with percentage green space cover (r^2^ = 0.16), overall NDVI (r^2^ = 0.20) and even more weakly with cover by individual vegetation types (r^2^ 0.03 – 0.09). This confirmed that our metric was not acting as a surrogate for vegetation extent (i.e. quantity) but as a reliable measure of the vigour of existing vegetation (i.e. a measure of quality). This approach has been used elsewhere to measure vegetation health and has proven to be effective in discriminating between levels of vegetation quality across urbanisation and socio-economic gradients [15].

### Spatial distribution of GI

**Fig. 2:**
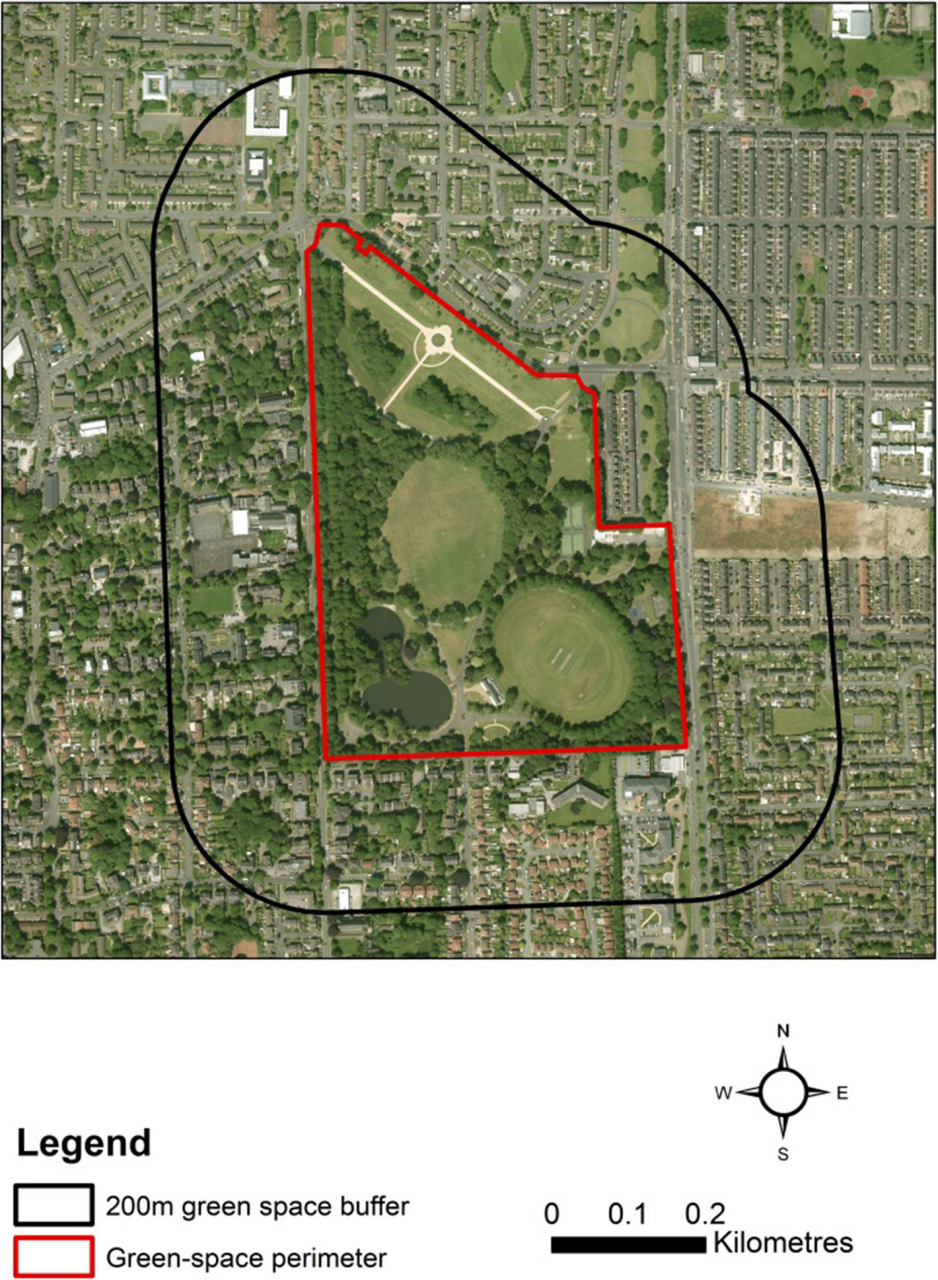
The measure of proximity used in this study. (Aerial imagery source: Edina [44]© Getmapping Plc)

A measure of the percentage population per LSOA living in close proximity to public parks and recreation land was calculated using high resolution (10 m) population data from the University of Southampton’s OpenPopGrid [41] dataset and the Ordnance Survey Open Green-space layer [42]. The latter comprises data on green-spaces accessible to the public in the United Kingdom. Three proximity buffers were created around accessible green space polygons in the Ordnance Survey Open Green-space layer. Firstly, buffers of 100, 200 and 300 metres (after European Commission guidelines, 2001) were created around green-space boundaries [43]. For each buffer, the values of population cells falling inside their boundary were summed and calculated as a percentage of the LSOA total. Green-space in the Ordnance Survey Open Greenspace layer were spatially co-incident with green-space within the public parks and recreation class in the ILM used in this study (see Table 3 and the original methodology covered in [18]) and therefore our metric represents the proportion of the local population in proximity to accessible recreational green spaces. Natural England (the UK government’s advisor on the natural environment in England) recommends a minimum size of 2 ha for local green-spaces and so a third variable, selecting only recreational green spaces over 2 ha, was created. Figure 2 gives an example of a 200 metre buffer measured from a green-space boundary.

### Socio-demographic and health data

Socio-economic and health data were obtained from the English Indices of Multiple Deprivation (IMD) 2015 [45]. This dataset provides scores for each LSOA in England on deprivation related to income, crime, health, employment, education, living environment and barriers to housing and services. Each deprivation domain is calculated from sub-domains which can be obtained in disaggregated form. The sub-domain indicator *Comparative Illness and Disability Ratio* (CIDR) of the Health and Disability Deprivation domain within the IMD dataset provides a measure of chronic morbidity at the neighbourhood level. This indicator is derived from the proportion of the local population suffering from chronic ill-health and is principally calculated from data on income benefits received as a consequence of long-term illness. The index is age- and sex-standardized thereby allowing us to make realistic comparisons between different demographic groups. The index is derived from a range of work and pension-related income benefits. Individuals can only claim one such benefit at a time and working-age and retirement-age claims are included [45]. Therefore the index is free from biases such as double counting and disproportionate representation by certain demographic groups. We chose to use an objective measure of population morbidity, rather than self-reported health or mortality (which together represent the most commonly used categories of indicator in studies on green-space and health [17]). This decision was taken in order to reflect the need to address the rise in non-communicable diseases (or the so-called epidemiological shift) which has accompanied increasingly urban, sedentary lifestyles as well as the changing urban demographics resulting from ageing human populations [46]. Of the health sub-domains in the IMD, the CIDR indicator correlates most closely with the over-arching health deprivation domain (*r*^2^ = 0.91). LSOAs in Greater Manchester had a mean CIDR score of 144 (SD 45) compared to 112 (SD 41) for all 32,844 LSOAs in England highlighting the relatively high levels of health deprivation in the city-region. Greater Manchester therefore provides a suitable study area to investigate the potential mitigation of population morbidity through the presence of green infrastructure. Data on neighbourhood (LSOA) population age ranges were downloaded from the UK Data Service for the UK 2011 census [43].

### Statistical analyses

Associations between GI variables and chronic morbidity were explored through multi-level linear regression analyses in a three-step process. In step one overall non-built land-cover was regressed on chronic morbidity stratifying by tercile groups for income (low, medium and high according to the IMD income deprivation field) and proportion of the adult population over sixty years of age, hereafter: younger neighbourhoods (0 – 17.3% (bottom tercile) adults ≥ 60 years), mid-age range neighbourhoods (17.4 – 23.6 % adults ≥ 60 years) and older neighbourhoods (> 23.6 % (top tercile) adults ≥ 60 years). In step two, a second series of models were developed where individual, disaggregated GI attributes (i.e. all land-cover types, all land-use types, landscape attributes (SHDI, patch size, effective mesh size and vegetation NDVI) and proximity variables) were added in a forward-stepwise fashion until a final best-fit model was established (based on the model r^2^ statistic). In step three, to test for the influence of land-cover and patch size associated with individual land-use types, mean percentage non-built cover and patch size (determined through OS MasterMap data, 2017) of gardens, institutional land, amenity and public parks and recreation classes (i.e. all major urban land-uses associated with GI) per LSOA were then entered into a final series of models as additional independent variables to those in step two. All models were adjusted for income, employment, crime, education, and barriers to housing and services deprivation. Moran’s I statistics were computed for step one models to test for the influence of spatial auto-correlation in model residuals which can violate assumptions of variable independence in ordinary least squares regression models. A summary description of predictor variables and their inclusion in the analysis is provided in Table 1.

**Table 1:** Description of GI predictor variables used in the multi-level linear regression analysis in this study

Predictor variable	Description	Expressed as:	Use in regression analysis
Total non-built land-cover	Aggregated proportion of land-cover that consists of vegetation or water.	% cover LSOAˉ^1^	Step 1
Water	Proportion of land-cover by water (water bodies and water courses)	% cover LSOAˉ^1^	Step 2 and 3
Ground layer vegetation	Proportion of land-cover by grass lawns and other ground flora	% cover LSOAˉ^1^	Step 2 and 3
Field layer vegetation	Proportion of land-cover by tall grasses, forbs and shrubs	% cover LSOAˉ^1^	Step 2 and 3
Canopy	Proportion of land-cover by trees	% cover LSOAˉ^1^	Step 2 and 3
Public parks and recreation	Proportion of land-use that includes public parks, sports grounds, playing fields, allotments and community gardens	% cover LSOAˉ^1^	Step 2 and 3
Amenity green-space	Proportion of land-use consisting of landscaped open spaces in residential or commercial areas with primarily aesthetic functions	% cover LSOAˉ^1^	Step 2 and 3
Private gardens	Proportion of land-use by private domestics gardens	% cover LSOAˉ^1^	Step 2 and 3
Peri-urban	Proportion of non-urban land-use, primarily consisting of arable land, grazed pasture and peat moorland outside of the main urban extent of the study area but within the Greater Manchester administrative boundary	% cover LSOAˉ^1^	Step 2 and 3
Previously developed land	Proportion of land previously subject to development and without any formal function at the time of data production	% cover LSOAˉ^1^	Step 2 and 3
Urban fabric	Proportion of all developed (built) land-use types and associated built infrastructure including residential, commercial, transport and industrial	% cover LSOAˉ^1^	Step 2 and 3
Informal urban greenery	Proportion of non-built land-cover occurring within the urban fabric class but without formal designated use (e.g. street trees, roadside vegetation).	% cover LSOAˉ^1^	Step 2 and 3
% population ≤ 300 m to public parks and recreation land	Proportion of the local population living within 300 m of any site in the public parks and recreation class (including sites within 300 m but in neighbouring LSOAs).	% population LSOAˉ^1^	Step 2 and 3
% population ≤ 200 m to public parks and recreation land	Proportion of the local population living within 200 m of any site within the public parks and recreation class	% population LSOAˉ^1^	Step 2 and 3
% population ≤ 100 m to public parks and recreation land	Proportion of the local population living within 100 m of any site within the public parks and recreation class	% population LSOAˉ^1^	Step 2 and 3
% population ≤ 200 m to 2 ha public parks and recreation land	Proportion of the local population living within 200 m of sites > 2 ha within the public parks and recreation class	% population LSOAˉ^1^	Step 2 and 3
Shannon’s Diversity Index (SHDI)	Metric indicating land-cover diversity	Dimensionless with values starting from 0 without limit (though rarely exceeding 4)	Step 2 and 3
Mean Patch Size	Mean area of all non-built patches	m^2^ LSOAˉ^1^	Step 2 and 3
Effective Mesh Size (Meff)	Measure of connectedness of non-built land-cover	m^2^ LSOAˉ^1^	Step 2 and 3
% non-built cover in gardens	Proportion of gardens that is vegetation or water	Percentage of total gardens cover LSOAˉ^1^ that is non-built	Step 3
% non-built cover in amenity green-space	Proportion of amenity green-space that is vegetation or water	Percentage of total gardens cover LSOAˉ^1^ that is non-built	Step 3
% non-built cover in public parks and recreation land	Proportion of public parks and recreation land that is vegetation or water	Percentage of total gardens cover LSOAˉ^1^ that is non-built	Step 3
% non-built cover in institutional land	Proportion of institutional land that is vegetation or water	Percentage of total gardens cover LSOAˉ^1^ that is non-built	Step 3
Mean patch size of gardens	Mean area of garden land-use patches	m^2^ LSOAˉ^1^	Step 3
Mean patch size of amenity green-space	Mean area of amenity green-space land-use patches	m^2^ LSOAˉ^1^	Step 3
Mean patch size of public parks and recreation land	Mean area of public parks and recreation land-use patches	m^2^ LSOAˉ^1^	Step 3
Mean patch size of institutional land	Mean area of institutional land-use patches	m^2^ LSOAˉ^1^	Step 3

## Results

Descriptive statistics related to areas characterised by younger, mid-age range and older neighbourhoods are given in Table 2. Characteristics related to percentage cover by land-use and land-cover are given in Table 3 and mean areas and percentage green cover of major land-uses are presented in Table 4.

**Table 2:** Descriptive statistics of age distributions within the areas characterised by having younger, mid-range and older populations

Age range	Younger neighbourhoods		Mid-age range neighbourhoods		Older neighbourhoods	
	Mean %	SD	Mean %	SD	Mean %	SD
**Age: 20–24**	10·44	9.00	6.38	1.52	5.11	1.19
**Age: 25–29**	10·41	6.09	6.93	1.80	4.89	1.51
**Age: 30–44**	22·89	4·33	21·28	2·23	18·27	2·28
**Age: 45–59**	15·69	4·56	19·86	2·67	21·34	2·45
**Age: 60–64**	3·78	1·38	5·88	1·10	7·61	1·54
**Age: 65–74**	4·92	1·64	7·81	1·16	11·53	2·35
**Age:75–84**	2·90	1·10	4·81	1·02	7·07	1·76
**Age:85–89**	0·69	0·41	1·27	0·53	1·80	0·77
**Age ≥ 60**	12·62	3·98	20·39	1·78	28·92	4·23

**Table 3:** Land-use and land-cover variables (NB urban fabric class includes informal urban greenery)

GI variable	Younger neighbourhoods						Mid-age range neighbourhoods						Older neighbourhoods					
	Low income		Medium income		High income		Low income		Medium income		High income		Low income		Medium income		High income	
	Mean	SD	Mean	SD	Mean	SD	Mean	SD	Mean	SD	Mean	SD	Mean	SD	Mean	SD	Mean	SD
**% All green-blue cover**	**47·3**	19·3	**47·5**	18·6	**49·1**	23·2	**55·9**	17·1	**57·9**	17·1	**65·5**	15·4	**52·5**	18·5	**63·4**	16·4	**69·3**	16·0
**%Water**	**0·8**	1·4	**1·3**	2·6	**1·9**	3·9	**1·1**	2·1	**1·0**	1·6	**1·3**	2·4	**1·0**	2·2	**1·3**	3·2	**1·4**	4·2
**%Ground layer**	**10·5**	6·2	**10·8**	6·0	**10·8**	7·4	**13·2**	6·0	**13·9**	6·7	**16·8**	7·2	**12·1**	6·5	**15·0**	7·3	**17·1**	8·2
**%Field layer**	**16·5**	10·8	**13·9**	9·6	**15·5**	13·3	**20·4**	10·3	**19·9**	11·6	**22·0**	13·6	**19·3**	10·5	**23·2**	11·4	**25·2**	13·2
**%Canopy**	**19·5**	8·3	**21·5**	8·9	**20·9**	11·7	**21·2**	8·2	**23·0**	9·9	**25·4**	8·9	**20·2**	8·5	**24·0**	9·6	**25·5**	9·7
**% Institutional land**	**3·5**	4·7	**3·6**	4·9	**3·1**	5·7	**3·6**	4·6	**3·0**	4·4	**2·4**	3·9	**2·8**	3·5	**3·5**	4·7	**2·5**	3·7
**% Public parks and recreation**	**9·6**	12·6	**10·5**	12·4	**7·7**	9·5	**8·5**	10·9	**12·2**	14·9	**9·6**	12·3	**8·5**	11·8	**10·4**	12·0	**9·7**	11·8
**% Amenity green-space**	**14·5**	10·8	**11·2**	12·0	**13·3**	12·7	**19·1**	13·7	**15·7**	13·3	**13·0**	12·9	**15·0**	12·2	**18·9**	14·7	**14·1**	13·1
**% Private gardens**	**27·6**	15·9	**27·8**	17·1	**24·8**	19·7	**27·2**	14·5	**31·3**	15·2	**38·1**	20·1	**27·9**	15·8	**30·0**	14·5	**36·0**	19·6
**% Peri-urban**	**6·3**	15.4	**8·7**	20·7	**11·3**	19·5	**11·7**	21·5	**9·8**	18·0	**15·6**	23·5	**12·6**	24·0	**15·8**	22·1	**21·7**	27·3
**% Previously developed land**	**0·7**	2·7	**0·6**	2·9	**1·1**	2·8	**0·6**	1·6	**0·3**	1·2	**0·2**	0·7	**0·3**	0·8	**0·2**	1·1	**0·3**	1·7
**% Urban fabric**	**37·8**	18·4	**37·6**	18·2	**38·8**	26·6	**29·5**	15·8	**27·7**	13·3	**21·0**	10·9	**32·9**	18·0	**21·2**	11·4	**15·8**	8·9
**% Informal urban greenery**	**7·9**	3·4	**8·4**	4·0	**7·8**	5·0	**7·7**	3·4	**7·3**	3·3	**7·5**	4·1	**7·8**	3·2	**6·1**	3·1	**5·7**	3·2
**% population ≤ 300 m to public parks and recreation land**	**90.6**	16.5	**89.9**	16.1	**77.8**	26.3	**87.3**	18.5	**88.8**	18.0	**80.8**	22.7	**90.8**	13.2	**85.2**	20.5	**75.7**	24.8
**% population ≤ 200 m to public parks and recreation land**	**72·0**	23·6	**72·3**	24·7	**57·2**	29·9	**66·0**	24·1	**69·8**	23·3	**59·5**	27·8	**70·2**	21·5	**65·5**	25.4	**53·1**	26·6
**% population ≤ 100 m to public parks and recreation land**	**34·6**	19·0	**36·9**	21·2	**25·8**	20·4	**31·1**	18·4	**36·2**	19·1	**27·9**	19·0	**35·6**	19·8	**33·1**	20·1	**24·1**	17·0
**% population ≤ 200 m to 2 ha public parks and recreation land**	**33·1**	29·6	**36·0**	31·9	**24·0**	29·5	**31·5**	28·4	**37·2**	29·9	**32·9**	30·2	**33·2**	25·0	**35·3**	29·5	**28·3**	26·4

**Table 4:** descriptive statistics of major urban green infrastructure types (based on OS Mastermap Greenspace Layer, 2017). Mean % green cover indicates mean proportion of each land-use that is green or blue space, i.e. non-built

Land-use	Low income				Medium income				High income			
Younger neighbourhoods	Mean % green cover	SD	Mean plot size (m^**2**^)	SD	Mean % green cover	SD	Mean plot size (m^**2**^)	SD	Mean % green cover	SD	Mean plot size (m^**2**^)	SD
Amenity	**76·1**	17·1	**491**	2178	**74·8**	18·8	**617**	3388	**74·5**	18·8	**712**	3812
Institutional	**49·7**	26·0	**356**	1211	**48·9**	25·9	**360**	1120	**49·6**	29·5	**417**	1860
Public parks and recreation	**76·1**	27·5	**1734**	7446	**79·1**	24·3	**1497**	4385	**79·4**	23·3	**1494**	5867
Private Garden	**41·7**	15·3	**77.0**	102	**43·1**	14·2	**82**	120	**42·3**	19·0	**123**	170
**Mid-age rage neighbourhoods**												
Amenity	**81·4**	13·8	**601**	3719	**82·8**	13·7	**797**	4217	**84·7**	15·1	**1224**	5454
Institutional	**55·9**	25·1	**386**	1429	**54·5**	26·1	**512**	2624	**58·6**	28·4	**553**	2156
Public parks and recreation	**80·5**	22·4	**1563**	6961	**85·6**	19·3	**2149**	10,104	**88·0**	17·8	**2243**	7657
Private Garden	**48·6**	13·7	**89**	113	**49·5**	11·8	**106**	153	**57·4**	13·0	**172**	243
**Older neighbourhoods**												
Amenity	**76·9**	13·9	**744**	5616	**86·8**	12·0	**1041**	5159	**88·7**	11·0	**1248**	5750
Institutional	**50·1**	25·5	**387**	1296	**62·5**	22·4	**495**	1931	**68·4**	21·9	**561**	2625
Public parks and recreation	**82·2**	21·0	**2242**	8036	**89·0**	13·9	**1890**	7214	**90·3**	15·5	**2383**	11,105
Private Garden	**48·9**	12·3	**94**	130	**51·6**	13·7	**138**	216	**58·3**	14·3	**227**	308

Overall GI quantity (green cover) increased with both income and age, with the lowest mean cover in low income, younger neighbourhoods (47·3%; SD 19·3) and highest mean cover in high income older neighbourhoods (69·3%; SD 16·0). Provision of public parks and recreation land and percentage of the population in close proximity to this land-use was generally greatest in middle income areas whereas amenity land was most abundant in lower income areas. Private gardens were greatest, in terms of extent, size and proportion of non-built cover, in higher income older neighbourhoods. Low income younger neighbourhoods exhibited the smallest mean garden size (77 m^2^ (SD 102) of which 41·7% (SD 15·3) was non-built cover) compared to older and higher income areas (mean size 227 m^2^ (SD 308) of which 58·3% (SD 14·3) was non-built cover, Table 4). Individual green land-cover types likewise exhibited greater extent in more affluent and older demographic groups.

Table 5 gives the results from step one of the analyses regressing green-space on the chronic morbidity with all green land-cover types aggregated into a single (negative ß values denote a positive association with better health).

**Table 5:** output from step one models: regressions of overall green cover on chronic morbidity (*, ** and *** indicate significance at *p* < 0·05, 0·01 and 0·001 respectively). Parameters exhibiting negative beta values imply an inverse association with area-level chronic morbidity and, therefore, predict better health

	Income level		
	Low	Medium	High
**Younger neighbourhoods**	ß	ß	ß
Income deprivation	0·167*	0·299***	0·426***
Barriers to Housing and Services Score	0·249***	0·657***	0·564***
Employment deprivation	0·584***	–	–
Crime	–	0·131**	0·291***
% Green cover	–	−0·17**	−0·234**
*r*^2^	0·69	0·69	0·65
Moran’s I	0.212***	0.072***	0.136***
**Mid-age range neighbourhoods**			
Income deprivation	0·303***	0·227**	–
Employment deprivation	0·487***	0·607***	0·754***
Barriers to Housing and Services	0·19***	0·075*	0·157**
% Green cover	−0·131**	–	–
*r*^2^	0·78	0·69	0·59
Moran’s I	0.163***	0.106***	0.497***
**Older neighbourhoods**			
Income deprivation	0·388**	0·209**	0·123***
Employment deprivation	0·531***	0·682***	0·82***
% Green cover	–	−0·162***	−0·138***
*r*^2^	0·80	0·78	0·72
Moran’s I	−0.089	0.239***	0.369***

The strongest associations between percentage non-built cover and health were seen in younger neighbourhoods of medium or high income. Analyses for these groups also produced the lowest model fit with highest model r-squared values exhibited by analysis of older age-group. Similarly, spatial-autocorrelation (Moran’s I tests on model residuals) exhibited variability across socio-demographic groups. Higher income groups were generally subject to a higher influence by spatial auto-correlation, particularly in mid-age range and older neighbourhoods. All other socio-demographic groups however exhibited low (Moran’s *I* < 0.3) or non-significant levels of non-stationarity in regression models, suggesting the analysis was robust to spatial effects and comparable to those found elsewhere [47]. Step two of the analysis produced generally better fitting models than step one as well as significant associations between individual GI attributes in cases where overall green-cover quantity (step one) proved to be non-significant. In this second step model fits (r-squared values) were generally higher in lower income models, though significant positive associations between health and GI attributes were most common in higher income areas in older neighbourhoods. In younger neighbourhoods, size and proximity to > 2 ha public parks and recreation land predicted better health in middle income areas. In low income areas vegetation quality (vNDVI) predicted better health whereas percentage cover by domestic gardens and institutional land predicted poor health. In the mid-age range population vegetation NDVI (in low and middle income areas), ground vegetation (in low income areas) and gardens (in high income areas) predicted lower chronic morbidity levels. In older neighbourhoods, a greater range of land-cover and informal urban GI was linked to better health in high income areas, whereas population in proximity to public parks and recreation land in low income areas (Table 6) was the strongest predictor of better health.

**Table 6:** regression outputs from step two models: regressing all landscape variables on chronic morbidity (*, ** and *** indicate significance at *p* < 0·05, *p* < 0·01 and *p* < 0·001 respectively)

		Younger neighbourhoods			Mid-age range neighbourhoods			Older neighbourhoods		
		Income level			Income level			Income level		
Parameter	All population model	Low	Medium	High	Low	Medium	High	Low	Medium	High
	ß	ß	ß	ß	ß	ß	ß	ß	ß	ß
**Income deprivation**	0·154***	0·138*	0·322***	0·397***	0·291***	0·216**	–	0·425**	0·205**	–
**Employment deprivation**	0·578***	0·633***	–	–	0·515***	0·617***	0·528***	0·492***	0·694***	0·654***
**Barriers to housing and services**	0·165***	0·222***	0·667***	0·562***	0·158***	0·091*	0·106*	–	–	0·094**
**Education, skills and, training deprivation**	0·065***	–	–	–	–	–	0·338***	–	–	0·176***
**Crime**	0·058***	–	0·139**	0·283***	–	–	–	–	–	–
**% Ground vegetation**	−0·041***	–	–	–	−0·110**	–	–	–	–	−0·099**
**% Field layer vegetation**	−0·045***	–	–	− 0·248**	–	–	–	–	−0·130**	–
**% Canopy**	−0·030***	–	–	–	–	–	–	–	–	−0·070*
**Domestic gardens**	−0·046***	0·064*	–	–	–	–	−0·120*	–	–	–
**Institutional land**	0·034***	0·074*	–	–	0·093**	–	–	–	–	–
**Informal urban greenery**	0·021*	–	–	–	–	–	–	–	–	−0·13***
**Mean patch size**	−0·041***	–	−0·239***	–	–	–	–	–	–	− 0·116***
**Vegetation NDVI**	−0·042***	− 0·12***	–	–	−0·071*	− 0·119**	–	–	–	–
**SHDI**	−0·036***	–	–	–	–	–	–	–	–	− 0·084**
**Population ≤ 200 m to public parks and recreation land ≥ 2 ha**	–	–	− 0·149**	–	–	–	–	–	–	–
**Population ≤ 100 m to public parks and recreation land**	–	–	–	–	–	–	–	−0·138*	–	–
***r***^**2**^	0·90	0·72	0·65	0·66	0·79	0·7	0·68	0·82	0·77	0·78

**Fig. 3:**
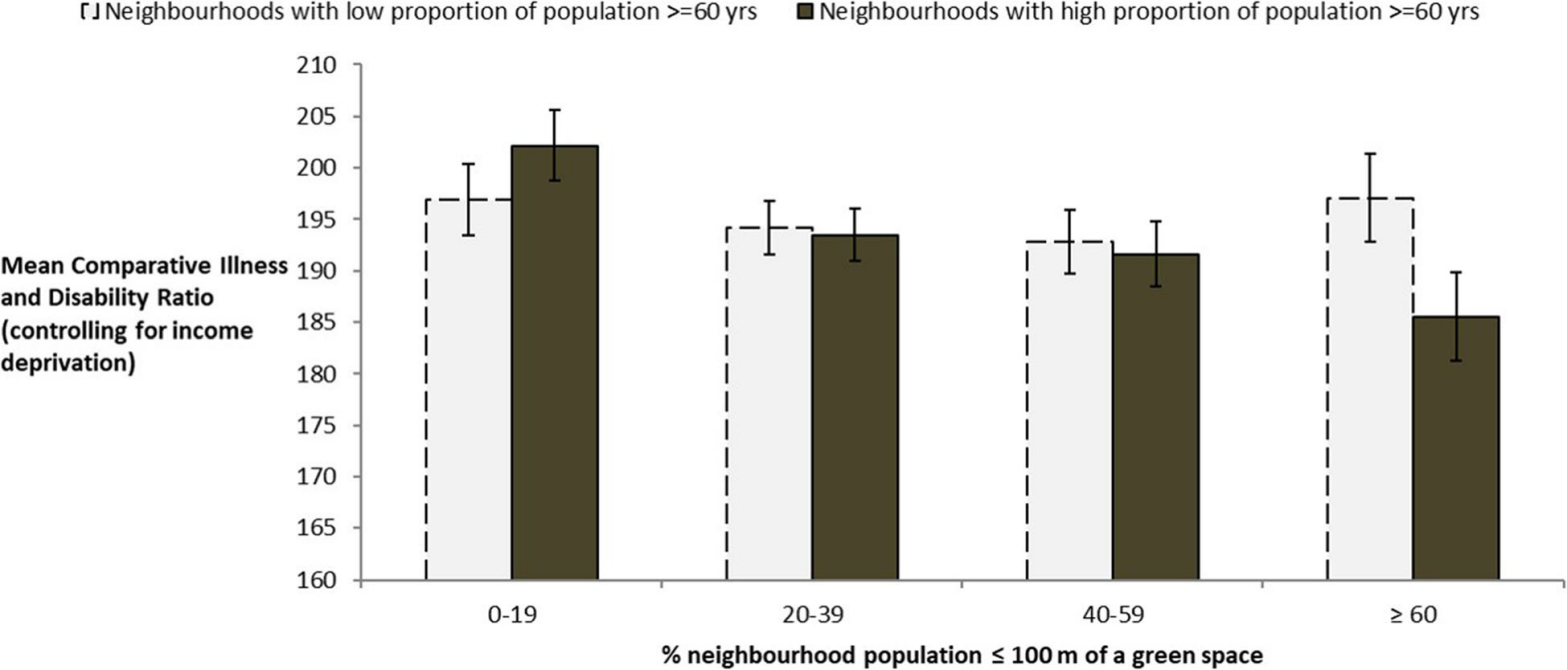
Mean levels of chronic morbidity (CIDR scores; error bars indicate 95% CIs) for older neighbourhoods (> 23·6% adults over 60 years of age) and younger neighbourhoods (≤ 23·6% adults over 60 years of age) grouped by percentage population within 100 m of public parks and recreation land, controlling for income deprivation (with standard error bars). Derived from the regression model for lower income areas in Table 6 in which only results for older neighbourhoods were significant (black bars). Note the CIDR indicator is age- and sex-standardized

Figure 3 gives mean CIDR scores or neighbourhoods with high (top tercile) versus low (bottom and middle terciles) adult population over 60 years of age across categories of percentage population in proximity to public parks and recreation land and shows that in areas characterised by having more older people, health decreases with distance from greenspace.

In the case of older neighbourhoods, a clear trend can be seen linking increasing population in proximity to public parks and recreation land and lower mean levels of chronic morbidity. In contrast, health outcomes for the same socio-demographic group did not exhibit associations with overall GI in step one models. This highlights the importance of considering individual GI types and their spatial distribution as predictors of health outcomes for different sectors of the urban population.

### Step three results

Entering GI variables related to size and proportion of non-built cover of land-uses had a significant bearing in the analysis. Percentage cover by gardens was not a significant predictor of better health when size and non-built cover in gardens were also entered in the model, with garden size exhibiting a positive association with health in the global (all population) model (β = − 0·089; *p* = < 0·001). However, in stratified models, garden size was only significant in high income older neighbourhoods (β = − 0·136; *p* = 0·001). The proportion of non-built land-cover in gardens was relevant in three cases: in high income mid-age range neighbourhoods (β = − 0·263; *p* < 0·001) and in middle (β = − 0·115; *p* = 0·003) and high (β = − 0·304; *p* < 0·001) income older neighbourhoods. Likewise, the proportion of non-built cover in amenity spaces was prominent in the all-population model (β = − 0·043; *p* < 0·001) but subsequently found to be significant only in middle income older neighbourhoods (β = − 0·144; *p* < 0·001). Model fits were generally higher when measures of size and the proportion of non-built cover of these land-uses were considered (see Supplementary Materials Table S1).

## Discussion

The proposal that older residents may benefit disproportionately from the protective effects of urban trees, hypothesized here and elsewhere [48] was supported by positive associations between tree canopy and better health within high income and older neighbourhoods. However, ground vegetation, SHDI, mean patch size and, in particular, informal urban greenery exhibited stronger associations suggesting that larger patches of green-space and diverse informal vegetation cover are more closely linked to better health in older neighbourhoods. Ground, canopy and field level vegetation showed different degrees of relevance across areas characterised by different socio-demographic groups confirming that the influence of land-cover types can vary as a function of socio-demographic context. This finding is significant given that previous health analyses have been limited to broad descriptions of green-space, or emphasized individual vegetation types in isolation [49, 50]. In contrast, we believe the current study to be the first to consider a range of land-use and land-cover characteristics employed across multiple stratified socio-demographic levels as part of a GI approach.

We found no significance between percentage cover by public parks and recreation spaces within LSOAs and chronic morbidity. However, when operationalized as the percentage of the population in close proximity to such spaces, significant positive associations with better health were observed. Indeed, this was the only significant GI predictor of health in low income older neighbourhoods. This supports our hypothesis that proximity to public parks and recreation land is disproportionately relevant to older age groups. This was, however, only the case in low income areas, in keeping with other work claiming that less affluent areas receive the greatest health benefits from the presence of urban green-space [8, 49]. A combination of older age and lower income in particular suggests low physical mobility and as a consequence, such neighbourhoods will be the most reliant on nearby public parks and recreation land.

Proximity to public parks and recreation land larger than two hectares showed a significant positive association with health in middle income younger neighbourhoods. LSOAs within this group exhibited the highest percentage of the population in close proximity to this land-use and the lowest overall mean patch size (data not shown), the latter also positively correlating with health for this group. It follows that in the most built up areas, large green spaces may act as potential buffers against chronic ill-health by providing opportunities for recreation [51] and relief from the physiological [52] and psychological [53] stressors associated with highly urbanised landscapes. Access to green-space > 2 ha has long been a recommendation associated with potential public health benefits (from Unwin’s memorandum in the 1920s and is now firmly part of the Natural England agenda) however, Pauleit et al. indicate that this is not being well implemented at a local authority level [54]. Peri-urban land, although exhibiting substantial and almost entirely non-built land-cover, was not significant in any of the models. This underlines the potential of nearby green spaces within the urban fabric of cities for reducing the health burden associated with chronic disorders prevalent in urban populations.

Percentage cover by amenity land-use demonstrated non-significant associations with health. This was perhaps surprising given that this land-use was generally most abundant in low income areas and that health outcomes in such areas in particular have been shown to be sensitive to GI cover [8, 49]. This suggests that the presence of green-space alone may not always be sufficient to bring about desired health outcomes for some groups, and supports assertions by other authors that quality in addition to quantity is an important factor for health outcomes [7, 17]. Unlike previous work employing measures of proximity and exposure to green-space [9, 55], this study also included indicators of size, extent of non-built-cover and quality which were particularly relevant to health. Vegetation health was a significant factor for three socio-demographic groups. Two of these were in low income groups with accompanying low measures of overall green cover, suggesting vegetation quality may become disproportionately significant in areas that are both income- and GI-deprived. In our study however, vegetation health did not appear to be a significant factor associated with health in neighbourhoods characterised by older populations. Rather we found that neighbourhoods with both low-income and a high proportion of older adults exhibited particular health associations with local accessible green-space. It is possible that where reliance on local nature is greatest (i.e. when both low income and increasing age restrict mobility), proximity is more relevant than quality for predicting better health.

Garden cover exhibited a positive relationship with health in high income, middle age-range populations but a negative association in the low income younger neighbourhoods. These two groups showed great disparity in terms of percentage cover by domestic gardens, with the latter exhibiting the highest mean cover for the study (Table 4). Similarly, garden green-cover was only significant within groups in which mean garden green cover was > 50%, and garden size was only a significant factor in the demographic group exhibiting the largest mean size (high income older neighbourhoods). The influence of domestic gardens on health may, therefore, be subject to threshold effects determined by their size and quality.

### Study limitations

This study had some limitations. Firstly, as a correlational study, we cannot rule out the effects of self-selection of healthier people into greener areas. However, the comprehensive range of GI variables used to quantify the natural environment and the stratified approach based on socio-demographic groups used here means that the effects of self-selection on the results are less likely. Secondly, the presence of ecological fallacy cannot be ruled out and further analysis at the individual level would be helpful to confirm the validity of our results. For example, we did not include any consideration of individual user preferences such how older people choose to use their leisure time from the point of view of nature engagement. Thirdly, it was not clear whether unique associations between environmental variables and socio-demographic characteristics occurred as a function of social or physical environmental processes. For example, ground vegetation, tree cover and SHDI were uniquely associated with better health in high income older neighbourhoods. Our approach cannot confirm, however, if this resulted from a particular affinity between this socio-demographic group and these characteristics or simply from particularly high mean values for these variables in such neighbourhoods. However, that other areas with similar mean values did not exhibit similar results suggests that socio-demographic factors may play at least a partial role in such associations.

Our GI dataset did not include other small-scale well established examples of urban green infrastructure such as bio-swales and green roofs that also provide important regulating functions (i.e. flood retention or reduced energy demand). Such technical ‘nature-based solutions’ [56] are included as green infrastructure from a strict planning perspective and would also be expected to have an influence on health and wellbeing. However, such features were difficult to identify with the existing methodology [18] and were therefore only included if identified as non-built cover. This limitation is acceptable as such nature-based solutions represent a very small proportion of GI provision. This study also had several strengths. We believe the characterization of the study area landscape to be the most comprehensive to date to be used in a cross-sectional ecological study of population chronic morbidity. This approach revealed important associations in socio-demographic groups where a coarse representation of overall green cover, as used extensively in previous studies [4, 7–9, 49], suggested none. Consequently we were able to identify individual land-use and land-cover associations relevant to area-level health. This is an important step if epidemiological findings are to be translated into urban planning and policy by allowing the latter to deliver evidence-driven interventions according to socio-demographic contexts and move beyond ‘one-size-fits-all’ approaches to green-space allocation. In addition, this is one of few studies to consider the influence of spatial autocorrelation on our analyses. Doing so revealed that this influence was within acceptable levels and particularly low or non-significant in neighbourhoods with high proportions of older people and those on low income. Our results are therefore robust to non-stationarity, especially in the case of socio-demographic groups carrying the highest health burden which were the focus of our study. Our approach using GI is also timely given its traction with planning practitioners in the UK and more widely [57], and provides an important foundation for delivering interventions for improving the health and wellbeing of urban dwellers.

## Conclusions

We took a GI approach to the analysis of associations between health (health deprivation) and the natural environment in urban areas. Our study considered the widest range of GI attributes yet examined, namely land-cover, land-use, landscape attributes (SHDI, patch size, effective mesh size and vegetation NDVI) and proximity variables. All of our proposed hypotheses were supported to some degree by the results of this study. Firstly, older people in low income urban areas appear to be disproportionately healthier if those areas are served by local accessible green spaces. Secondly, the quality (vegetation health) as well as quantity (percentage cover by vegetation) associated with urban green infrastructure predicted health outcomes. Attributes such as size, non-built cover and land-cover diversity likewise appeared to be relevant to our analysis of patterns in neighbourhood-scale chronic morbidity. In addition, our over-arching supposition that associations between health and GI are moderated by socio-demographic contexts was upheld. An emphasis on publicly accessible GI, maintenance of healthy vegetation and diverse, nearby green spaces represent promising opportunities for public health interventions particularly in areas characterised by low income and high proportions of older people. These findings have direct consequences for urban planning and may help to inform international initiatives, such as the World Health Organisation’s Age-Friendly Cities programme.

## Supplementary information

**Additional file 1.**

## Data Availability

The green infrastructure dataset used in this study is available on request from the corresponding author but restrictions apply as it contains data under licence from the Ordnance Survey. Population data used in this study are freely available from the University of Southampton’s OpenPopGrid service: http://openpopgrid.geodata.soton.ac.uk/ Deprivation data are freely available from the UK government statistical service via: https://www.gov.uk/government/statistics/english-indices-of-deprivation-2015 LSOA level data on which statistical analyses are based will be available through the Natural Environment Research Council’s Environmental Information Data Centre (http://eidc.ceh.ac.uk/) upon publication of the results.

## Abbreviations

GI: Green infrastructure
ILM: Integrated landscape map
LSOA: Lower super output area
SHDI: Shannon’s diversity index
NDVI: Normalized difference vegetation index
IMD: Indices of multiple deprivation
CIDR: Comparative illness and disability ratio
SD: Standard deviation
