# Supplementary material for: Relationships between health outcomes in older populations and urban green infrastructure size, quality and proximity
Source: BMC Public Health. 2020 May 6;20:626. doi: 10.1186/s12889-020-08762-x (PMC7201616; doi:10.1186/s12889-020-08762-x)
Supplement: Supplementary file 1 — Additional file 1. [file 12889_2020_8762_MOESM1_ESM.docx]

**Relationships between health outcomes in older populations and urban green infrastructure size, quality and proximity: Supplementary Materials**

Table S1 gives the model outputs of step three of the analysis, with mean area and non-built cover of amenity, public parks and recreation spaces, and domestic gardens entered as additional variables into the regression models.

**Table S1 Step three regression model outputs (*, ** and *** indicate significance at *p* < 0·05, *p* < 0·01 and *p* < 0·001 respectively)**

|  |  | **Younger neighbourhoods** | | | **Mid-age range neighbourhoods** | | | **Older neighbourhoods** | | |
| --- | --- | --- | --- | --- | --- | --- | --- | --- | --- | --- |
|  |  | **Income level** | | | **Income level** | | | **Income level** | | |
| **Parameter** | **All population model** | **Low** | **Medium** | **High** | **Low** | **Medium** | **High** | **Low** | **Medium** | **High** |
|  | ß | ß | ß | ß | ß | ß | ß | ß | ß | ß |
| Income deprivation | 0·117** | 0·138* | 0·323*** | 0·380*** | 0·318*** | 0·237** | - | 0·405** | 0·254** | - |
| Employment deprivation | 0·587*** | 0·634*** | - | - | 0·484*** | 0·591*** | 0·445*** | 0·512*** | 0·625*** | 0·612*** |
| Barriers to housing and services | 0·191*** | 0·221*** | 0·668*** | 0·552*** | 0·183*** | 0·093* | 0·175*** | - | - | 0·159*** |
| Education, skills and, training deprivation | 0·059** | - | - | - | - | - | 0·249** | - | - | - |
| Crime | 0·056*** | - | 0·142** | 0·294*** | - | - | 0.170** | - | - | 0.063* |
| % Ground vegetation | -0·140* | - | - | - | - | - | - | - | - | - |
| % Field layer vegetation | - | - | - | -0·241** | - | - | - | - | - | - |
| % Canopy | - | - | - | - | - | - | - | - | - | 0.056* |
| Domestic gardens | - | 0·065* | - | - | - | - | - | - | - | - |
| Institutional land | 0·034*** | 0·076* | - | - | 0·077** | - | - | - | - | - |
| Informal urban greenery | - | - | - | - | - | - | - | - | - | -0·080** |
| Mean patch size | -0·029** | - | -0·240*** | - | - | - | - | - | - | - |
| Vegetation NDVI | -0·041*** | -0·125*** | - | - | -0·081* | -0·121** | - | - | - | - |
| SHDI | -0·025** | - | - | - | - | - | - | - | - | -0·088** |
| Population ≤ 200 m to public parks and recreation land ≥ 2 ha | -0.025** | - | -0·146** | - | -0.122 | - | - | - | - | - |
| Population ≤ 100 m to public parks and recreation land | - | - | - | - | 0.103 | - | - | -0·131* | - | - |
| Non-built cover in amenity spaces | -0.044*** |  |  |  |  |  | - |  | -0.146*** |  |
| Non-built cover in gardens |  |  |  |  |  |  | -0.263*** |  | -0.115** | -0.304*** |
| Garden area | -0.089*** | - | - | - | - | - | - | - | - | -0.136** |
| *r*² | 0·91 | 0·71 | 0·65 | 0·66 | 0·79 | 0·69 | 0·72 | 0·82 | 0·79 | 0·82 |
